# Churches and Charity Funds

**Published:** 1917-01-20

**Authors:** 


### CHURCHES AND CHARITY FUNDS.

Our article in The Hospital of the 6th inst. on "Immoralities in Church Management" has attracted a good deal of attention. If we may judge by the articles which have appeared in the Church papers, and the letters which have reached us, there is every hope that steps may be taken with a view to the adoption of some uniform practice.

On December 31, at the collections for the Red Cross, various plans seem to have been adopted where the total collection was squeezed for other purposes. We have evidence that in one church, on the Sunday preceding the day of collection, it was officially announced that next Sunday's collection would be for the Red Cross funds. When Red Cross Sunday arrived it was announced that for special reasons, which were not given, it had been decided not to hold collections for the Red Cross on that day, but to make collections for church expenses, postponing the Red Cross collection for five or six weeks. Members of the congregation, like loyal citizens, had brought their money for the Red Cross, and much indignation was felt and expressed. In another case, the Red Cross appeal was not announced until immediately before the collection; that in the morning being subject to deduction, and that in the evening being wholly for church expenses with boxes at the doors for Red Cross gifts. We have evidence that neither of these announcements was acted upon in fact, but a sum of £3 was deducted from the total collections and devoted to the parish room.

In some places of worship it is the custom to have the ordinary collection for the church's own needs every Sunday in the middle of the service, and when appeals are made for Special Objects the collection for them is made at the doors as the congregation leaves the building. Our contemporary the *Church Times*, while considering our former article too harsh, admits that, if deduction is made, without intimation that the money which the worshippers believe themselves to be giving to the special charity will be tapped, there is nothing to justify such action, and we were entitled to condemn it. The *Christian World* expresses agreement with this view too. (*See also page* 229.)

There is the further point as to what practice should be pursued on National Occasions like the collections for the Red Cross in which the whole nation is interested, and on Hospital Sunday. The principle of deductions from church collections, irrespective of the objects for which they are made, is wrong. In the few places where such a practice may prevail it would be better, in our opinion, for the authorities to reduce the number of collections for outside objects until business management had provided adequate funds to meet the church expenses. We have had experience in raising money through the churches, and hold to the opinion that where the place of worship is really the House of God, and so recognised and valued by the congregation, there every expense connected with its upkeep and maintenance can be readily met. That is why the majority of places of worship are free from the abuse of making deductions from church collections, irrespective of the objects for which they are made. Such practices are opposed to the feelings and conscience of a multitude of people attending places of worship, who resent the practice, and regard it as indefensible and wrong.

We should like to see the matter taken up by the Council of the Metropolitan Hospital Sunday Fund and by Sunday Fund Councils generally throughout the country. These Councils consist of representatives of all denominations, and it should not be difficult for a wise solution to be found by agreement, that, collections on National Occasions and on Hospital Sunday should be free from all deductions whatever. If and where the managers responsible for the upkeep of a place of worship have established a practice to make a collection at every service for its maintenance, that collection should be taken in the middle of the service, any collection for a Special Object being made after the sermon, at the doors, when the congregation leaves the church. Those places of worship, where an objectionable system of collection is practised, should not be given the privilege to make a collection for such objects as the Red Cross and the Hospital Sunday Fund. Is it not possible to remove all cause of grievance to members of the congregation, and of loss to the churches through the withdrawal of their members, by the adoption of some such simple modifications as we have indicated?

